# Identification of Four Secreted Aspartic Protease-Like Proteins Associated With Sophorolipids Synthesis in *Starmerella bombicola* CGMCC 1576

**DOI:** 10.3389/fmicb.2021.737244

**Published:** 2021-09-14

**Authors:** Jun Liu, Guoqin Zhao, Xinyu Zhang, Xin Song

**Affiliations:** ^1^State Key Laboratory of Microbial Technology, Shandong University, Qingdao, China; ^2^National Glycoengineering Research Center, Shandong University, Qingdao, China

**Keywords:** *Starmerella bombicola*, aspartic protease-like protein, acidic sophorolipids, ammonium sulfate, different nitrogen sources

## Abstract

The non-pathogenic yeast *Starmerella bombicola* CGMCC 1576 is an efficient producer of sophorolipids (SLs). The lactonic SLs are mainly produced with yeast extract, and the acidic SLs are mainly produced with ammonium sulfate. Naturally produced SLs are a mixture of various lactonic and acidic SLs. Usually, the SL mixture is not well separated technically, and the separation cost is relatively high. In order to reduce the cost of separation, four secreted aspartic protease-like proteins were identified through proteomic analysis of fermentation broth of *S. bombicola* under different nitrogen source conditions. The coding genes of the four proteins, namely, *sapl1*, *sapl2*, *sapl3*, and *sapl4*, are of high sequence similarity (above 55%) and included in a gene cluster. The expression of the four genes was significantly upregulated on (NH_4_)_2_SO_4_ compared with that on yeast extract. The four genes were deleted together to generate a strain Δ*sapl*. The titer of SLs in Δ*sapl* reached 60.71 g/L after 5 days of fermentation using (NH_4_)_2_SO_4_ as the nitrogen source and increased by 90% compared with the wild-type strain. The concentration of acidic SLs was 55.84 g/L, accounting for 92% of the total SLs. The yield of SLs from glucose (g/g) by Δ*sapl* was 0.78, much higher than that by wild-type strain (0.47). However, no increase of SLs production was observed in Δ*sapl* under yeast extract condition. Compared with that of the wild-type strain, the expression levels of the key genes for SLs synthesis were all upregulated to varying degrees in Δ*sapl* under (NH_4_)_2_SO_4_ conditions, and particularly, the expression level of *ugta1* encoding UDP glucosyltransferase was upregulated by 14.3-fold. The results suggest that the *sapl* gene cluster is negatively involved in the production of SLs in the case of (NH_4_)_2_SO_4_ by restraining the expression of the key genes involved in SLs synthesis. The Δ*sapl* strain is an excellent producer of high-titer and high-yield acidic SLs.

## Introduction

Biosurfactants are amphiphilic microbial molecules with hydrophilic and hydrophobic moieties. Biosurfactants have drawn more and more attentions of different industries due to their great advantages than their petroleum-based chemically synthesized counterparts, such as structural diversity, extensive foaming activities, low toxicity, greater biodegradability, bioavailability, biocompatibility, ecological acceptability, environmental friendliness, ability to function in wide ranges of pH, temperature, and salinity, as well as greater selectivity and lower critical micelle concentration (CMC). In addition, they exhibit good application prospects in the fields of antibacterial, antiviral, antitumor, and other pharmacology and immunity (Singh and Cameotra, 2004; Santos et al., 2016; Gaur et al., 2019; Valotteau et al., 2019).

Sophorolipids (SLs) are an important biosurfactant with the highest yield among all the biosurfactants and will be a sustainable substitute for chemical surfactants (Andersen et al., 2016). SLs are composed of hydrophilic sophorose molecule and hydrophobic long-chain hydroxy fatty acid linked by glycosidic bond. The key enzyme genes of the SLs synthesis have been revealed to be grouped in one large subtelomeric gene cluster (Van Bogaert et al., 2013; Anjum et al., 2016). The synthesis of SLs begins with the hydroxylation of fatty acids under the catalysis of cytochrome P450 family Cyp52m1 (Van Bogaert et al., 2009), the acidic SLs were formed by the catalysis of two UDP glucosyltransferases (Ugta1 and Ugtb1), and the substrates of two UDP glucose (UDPG) were produced by glucose metabolism (Saerens et al., 2011a,c). Under the action of acetyltransferase (Slat) (Saerens et al., 2011b), acidic SLs were transformed into form acetylated acidic SLs and released to the outside of cells under the transport of MDR protein (Liu et al., 2020). Besides the key enzymes responsible for SLs synthesis, some other enzymes closely related to SLs synthesis have successively been identified and characterized. Ciesielska et al. found that lactonase (Sble) can catalyze the conversion of acidic SLs to lactonic SLs (Ciesielska et al., 2016). A monooxygenase MoA that was related to the degradation of a specific SL molecule C18:2 DASL (di-acetylated acidic SL with a C18:2 fatty acid) has been identified and characterized (Li et al., 2016a); soon afterward, Li et al. (2016b) also discovered a long-chain acyl-CoA synthetase (Alcs), which is associated with the uptake of long-chain fatty acids. Recently, a protein Bro1 essential for SLs synthesis in *Starmerella bombicola* was revealed (Liu et al., 2020).

SLs are secondary metabolites secreted by *S. bombicola* under conditions of limited nitrogen sources. When there are only hydrophilic carbon sources (such as glucose and glycerol) in the medium, they will not be degraded to produce acetyl-CoA for growth and metabolism. In addition, part of glucose will be converted into UDPG, and fatty acids need to be synthesized *de novo*. When only hydrophobic substrates (such as hydrocarbons, fatty acids) are present, they are used for SLs synthesis after being activated. In addition, some of them are used to synthesize UDPG. When two carbon sources exist at the same time, the synthesis of SLs will be strongly stimulated. In laboratory studies, organic reagent extraction is used for the separation of SLs. For large-scale industrial applications, physical methods are used to separate SLs. The lactonic SLs will naturally deposit to the bottom of the broth at the end of fermentation. SLs can be determined by the sulfuric acid-anthrone method or HPLC analysis. HPLC-MS is used to analyze the structure of SLs. It is also possible to use preparative HPLC to obtain single-component SLs and then perform mass spectrometry analysis. SLs have good surface activity. SLs can reduce the surface tension of water from 72.8 mN/m, and their CMC is 11–250 mg/L (Develter and Lauryssen, 2010).

SLs produced by *S. bombicola* fermentation are a mixture of more than 20 different SL molecules and are grouped into two forms, namely, lactonic SLs and acidic SLs, based on whether the carboxyl group of the hydrophobic side chain in the molecule and the 4" hydroxyl group of the hydrophilic sophorose group form a lactone bond (Van Bogaert et al., 2011). Lactonic SLs exhibit good ability to reduce the surface interfacial tension, as well as good biological activities including antibacterial, antiviral, and anti-cancer, while acidic SLs have better water solubility and surface activity than lactonic SLs, which make acidic SLs be more favorable for many applications. The researchers also found that acidic SLs with high water solubility were more effective than lactonic SLs in enhancing repair of heavy metal-contaminated soil. Acidic SLs harbor low-foaming ability, water solubility, and remarkably low toxicity and have been utilized as low-foaming detergents for washing machines, such as laundry machines, and washer–disinfectors for cleaning medical devices (Hirata et al., 2009). Recently, Qi et al. (2018) used SLs produced by the fermentation of *S. bombicola* to study the removal capacity of cadmium and lead in contaminated soil and found that the removal rate of cadmium and lead by 8% crude acidic SLs reached 83.6 and 44.8%, respectively, and acidic SLs with high water solubility were more effective than lactonic SLs in enhancing remediation of heavy metal-contaminated soils.

The first step after fermentation is to separate acidic SLs from lactonic ones so that they can be further used for different purposes. Organic solvent extraction process is most commonly used in the separation process, which increases not only the separation cost but also the environmental burden. High proportion of lactonic or acidic SLs in SL mixture can be achieved by genetically engineering strains or changing the medium ingredients, especially the nitrogen sources (Ma et al., 2011; Roelants et al., 2016). A large amount of lactonic SLs is produced by fermentation of *S. bombicola* with yeast extract as the nitrogen source. Acidic SLs account for the vast majority in the SL mixture when ammonium sulfate was used as the nitrogen source; however, the titer of total SLs was relatively low (47.6 g/L) in the case of ammonium sulfate.

It is challenging to find some factors leading to high production and high proportion of acidic SLs in total SLs. The extracellular proteins of *S. bombicola* in the fermentation broth under two different nitrogen source conditions were analyzed, and four proteins were detected only in the presence of ammonium sulfate. Four genes encoding these proteins were identified as *sapl1*, *sapl2*, *sapl3*, and *sapl4* by sequence alignment. The aim of this work was to figure out whether the four proteins led to the differential synthesis of SLs under ammonium sulfate and yeast extract conditions and if it does further uncover the role of the four proteins and the probable mechanism by which they affect SLs synthesis.

## Materials and Methods

### Chemicals and Reagents

The chromatographic grade acetonitrile and methanol were all purchased from TEDIA Company Inc. (Fairfield, CT, United States). Anthrone was purchased from Sigma (St. Louis, MO, United States). The antibiotics were purchased from Amresco (Solon, OH, United States). Agar powder, galactose, peptone, sorbitol, and other reagents were of analytical grade and purchased from Dingguo (Beijing, China).

### Yeast Strains and Culture Medium

SLs-producing strain *S. bombicola* CGMCC 1576 was isolated from sewage in our laboratory and preserved in China General Microbiological Culture Collection Center (CGMCC), numbered 1576 (Li et al., 2016b). *S. bombicola* CGMCC 1576 was used as a wild-type strain in this research. *Pichia pastoris* GS115 was deposited in a −80°C ultra-low temperature refrigerator by our laboratory for heterologous protein expression. The SC (yeast synthetic medium) contained (g/L): yeast nitrogen base 1.7, (NH_4_)_2_SO_4_ 5.0, yeast synthetic drop-out medium supplements 1.3, and glucose 20.0 (galactose-induced medium in which glucose is replaced by galactose). The fermentation medium contained (g/L): glucose 80.0, oleic acid 60.0, yeast extract 3.0 or (NH_4_)_2_SO_4_ 1.4, KH_2_PO_4_ 1.0, Na_2_HPO_4_⋅12H_2_O 1.0, and MgSO_4_⋅7H_2_O 0.5. The BMGY medium contained (g/L): yeast extract 10.0, peptone 20.0, K_2_HPO_4_⋅3H_2_O 3.0, KH_2_PO_4_ 11.8, yeast synthetic drop-out medium supplements 3.4, (NH_4_)_2_SO_4_ 10.0, 1% (v/v) glycerin, pH 6.0, and 2 ml 0.82 mM biotin was added after sterilization. The BMMY medium contained (g/L): yeast extract 10.0, peptone 20.0, K_2_HPO_4_⋅3H_2_O 3.0, KH_2_PO_4_ 11.8, yeast synthetic drop-out medium supplements 3.4, (NH_4_)_2_SO_4_ 10.0, pH 6.0, and 2 ml 0.82 mM biotin and 1% methanol were added after sterilization. Firstly, strains were cultured overnight in liquid YEPD medium at 30°C at 200 rpm until the biomass concentration measured at 600 nm was approximately 1.0, and then 2% (v/v) of the above cell cultures were inoculated into 50 ml of fermentation medium in a 300 ml shake flask and grown at 30°C, 200 rpm.

### Bioinformatics Analysis of Sapl Protein

The amino acid sequences were compared and analyzed using the online analysis tool BlastP.^1^ Alignment analysis of local genome sequence was edited using the BioEdit software, the phylogenetic tree was constructed by MEGA5, and then ClustalX was used to perform multiple sequence alignment on the homologous sequences.

### Proteomics Analysis of Fermentation Broth Under Different Nitrogen Sources

*S. bombicola* was fermented under two different nitrogen sources (yeast extract or ammonium sulfate). Under the condition of ammonium sulfate as the nitrogen source, acidic SLs are predominantly produced (Ma et al., 2011). To figure out the probable reason why the nitrogen sources yeast extract and ammonium sulfate caused differential synthesis of SLs, the proteome of the fermentation broth of *S. bombicola* under the two nitrogen sources was analyzed, and the proteins related to SLs synthesis were expected to be found. SLs are secondary metabolite and generally start synthesis after 3 days of cultivation. A large amount of synthesis happens on the 5th day, and the production stabilized on the 7th day. The fermentation broth after 7 days of cultivation was taken for extracellular proteins analysis. Trichloroacetic acid (TCA) precipitation method was used to extract the total protein and prepare the sample.

An equal volume of acetone solution was added to the fermentation broth. After mixing, the sample was centrifuged at 8,000 rpm for 20 min to discard the supernatant. The precipitate was washed with 80 and 100% acetone under magnetic stirring for 20–30 min and centrifuged at 8,000 rpm for 20 min. Then, the precipitate was dried to obtain dry powder. After the sample was denatured, reduced, and alkylated, trypsin was added (mass ratio 1:50) and enzymatically hydrolyzed at 37°C for 20 h. The enzymatic hydrolysis product was desalted, lyophilized, redissolved in 50% ACN/0.1% formic acid (FA) solution, and stored at -20°C until use. Q Exactive (Thermo Fisher Scientific) was used as a mass spectrometer. Solution A was an aqueous solution of 0.1% FA, and solution B was an aqueous solution of 0.1% FA in acetonitrile (84% acetonitrile). After the chromatographic column was equilibrated with 95% A liquid, the sample was loaded onto the Trap column by the autosampler. After each full scan, 20 fragmentation maps (MS2 scan) were collected. Finally, the raw file of the mass spectrometry test was used to search the corresponding database with the Proteome Discoverer 1.4 software, and finally, the result of the identified protein was obtained. Label-free, non-quantitative methods were used for the analysis of complex mass spectrometry data of extracellular proteins (Griffin et al., 2010).

### Transcription Level Analysis of *sapl* Genes Under Different Nitrogen Sources

Wild-type strain was cultivated in fermentation medium in triplicate under different nitrogen sources (yeast extract or ammonium sulfate). Samples were taken every 12 h until 5 days of cultivation. The cells were centrifugated for 5 min at 4°C, 5,000 rpm and stored in an ultra-low temperature refrigerator at −80°C. The total RNA extraction of the sample refers to the previous description with the RNAiso Plus reagent (TaKaRa, Japan) (Liu et al., 2020). The RNA sample was reverse transcribed into cDNA, and qRT-PCR was performed using LightCycler^®^ 480 system (Roche, Mannheim, Germany) and the SYBR^®^ Premix Ex Taq^TM^ (Prefect Real Time) (TaKaRa, Otsu, Japan). The actin filament gene *actin* (GenBank ID: KT002360) was used as the internal reference gene.

### Construction of Knockout Mutants

The hygromycin self-deleting expression cassette constructed by our laboratory (Li et al., 2016b) was used to recycle hygromycin marker in the later genetic manipulations. The coding genes of four extracellular acidic protease-like proteins were knocked out in sequence, and four gene-deleted strains were obtained using the principle of homologous recombination and named as Δ*sapl*. All primers used in this work are listed in Supplementary Table 1.

### Shake Flask Fermentation and Determination of the Amount of SLs

The four gene-deleted Δ*sapl* strains and wild-type strain were transferred from a glycerol tube to a 5 ml shaker tube containing 20.0 g/L glucose, 20.0 g/L peptone, and 10.0 g/L yeast extract at 30°C, 200 rpm overnight, until reaching the mid-log phase. Then, 2% (v/v) of the culture broth was transferred into 50 ml fermentation medium in 300 ml shake flasks and incubated at 30°C, 200 rpm. Samples for determination of dry cell weight, glucose, and SLs were collected on the fifth and seventh days of cultivation.

Two volumes of n-butanol/ethanol/chloroform (10:10:1) was added into the fermentation broth. After shaking and extraction, the mixture was centrifuged at 8,000 rpm for 10 min at room temperature. The cell pellets were washed twice with distilled water and then dried to a constant weight at 50°C (Liu et al., 2020). The fermentation broth was centrifuged for 10 min at 8,000 rpm to collect the supernatant. After the supernatant was properly diluted, 25 μl of the sample was used for determination of residual glucose by a biosensor SBA-40C (Shandong Academy of Sciences, Jinan, China) (Li et al., 2016a). The titer of SLs was measured using the anthrone sulfate method (Buschmann and Wodarczak, 1995; Li et al., 2016b).

### Growth Assay

Δ*sapl* and wild-type strains were grown in YEPD liquid medium at 30°C, 200 rpm overnight, respectively. Then, 2% (v/v) of the culture broth was transferred into 50 ml fermentation medium with yeast extract or ammonium sulfate as nitrogen sources in 300 ml shake flasks and incubated at 30°C, 200 rpm. Samples were taken every 24 h to determine the biomass, residual glucose, and pH of the fermentation broth. One milliliter of n-butanol/ethanol/chloroform (10:10:1) was added to 0.5 ml of the fermentation broth, mixed, and centrifuged at 10,000 rpm for 1 min at room temperature, and then the supernatant was removed. The precipitate was washed twice with distilled water and diluted with distilled water to an appropriate concentration for OD_600_ measurement using Epoch 2 Microplate Spectrophotometer (BioTek Instruments, United States). The pH value of the fermentation broth was measured with precision pH test paper.

### Heterologous Expression and Purification of the Sapl1 Protein

The Sapl1 protein was heterologously expressed in strain *P. pastoris* GS115. The coding sequence of the Sapl1 was amplified and purified from *S. bombicola* by using primer pair containing *Avr*II and *Not*I restriction sites, and 6xHis-tag sequence was inserted before the stop codon of the reverse primer. The pPIC9K plasmid was digested and recovered with *Avr*II and *Not*I. The *sapl*1 sequence and the digested plasmid were ligated in Solution I in a metal bath at 16°C for 4 h and then transferred to *Escherichia coli* DH5α, and positive transformants were picked and verified by sequencing. The pPIC9K-*sapl*1 plasmid was linearized by single restriction digestion and transformed into *P. pastoris* GS115 by electroporation. The correct transformant was inoculated in 5 ml liquid YEPD in test tubes and cultured at 30°C, 200 rpm overnight, and then 1% (v/v) of the broth was transferred into 50 ml BMGY liquid medium, with 2‰ of 0.82 mM biotin added, and cultured at 30°C, 200 rpm for 16–18 h. The precipitate was collected by centrifugation at 2,500 rpm for 5 min, and the cells were resuspended in BMMY medium and poured back into 100 ml of BMMY medium. Then, 2‰ of 0.82 mM biotin and 1% anhydrous methanol were added to the above BMMY medium and incubated at 30°C, 200 rpm, 1% anhydrous ethanol was added every 24 h, and the fermentation was ended after 6 days of cultivation. The supernatant of the fermentation broth was collected by centrifugation at 10,000 rpm, 4°C for 10 min, the Sapl1 protein was purified using a nickel column, and then a 10 kDa dialysis bag was used for desalination. The protein concentration was determined by Bradford Protein Assay Kit (Sangon Biotech, Shanghai, China) and verified by SDS-PAGE.

### Characterization of the Sapl1 Protein

Sapl protease activity was measured using the casein method, referring to the GB/T 28715–2012, determination of acidic and neutral protease activities in feed additives spectrophotometric method. The reaction was as follows: 1.0 ml of the diluted enzyme solution was added to a 10 ml test tube with a stopper, the test tube was placed in a 40°C water bath for 5 min, 1.0 ml of the same preheated 1% casein solution (pH 3.0 and 7.2, respectively) was added to the sample tube for 10 min, 2 ml 0.4 M TCA solution was added to all test tubes, and 1.0 ml 1% casein solution was added to the control tube and shaken well. All test tubes were placed in a 40°C water bath for 10 min, removed, quickly cooled to room temperature, and filtered with medium-speed qualitative filter paper.

One ml of the above-mentioned corresponding filtrate was pipetted into a new 10 ml test tube with a stopper, with 5.0 ml 0.4 M sodium carbonate solution and 1.0 ml dilute Folin-phenol reagent added, and the above mixture was incubated at 40°C for 20 min and then quickly cooled to room temperature. The concentration of tyrosine was determined by measuring the absorbance value at 680 nm. The enzyme activity formula was calculated as follows.


$$Xi=\frac{\left(c-c0\right)\times V\times 4\times N}{1.0\times m\times 10}$$

*X*_*i*_: protease activity (U/ml);

*c*: the concentration of tyrosine in the sample tube (μg/ml);

*c*_0_: the tyrosine concentration of blank tube (μg/ml);

*V*: total volume of the liquid enzyme sample (ml);

4: the total volume of the enzyme reaction system (ml);

*N*: the multiple of the sample extract;

1.0: the amount of enzyme involved in the reaction (ml);

*m*: the sample volume (ml);

10: response time (min).

### Nucleotide Sequence and Proteomics Data Accession Number

The GenBank accession numbers for the genes were as follows: *gme2461*, MW384876; *gme2462*, MW384877; *gme2463*, MW384878; and *gme2464*, MW384879. The mass spectrometry proteomics data were available *via* ProteomeXchange with identifier PXD023214.

## Results

### Bioinformatic Analysis of Sapl

As previously reported (Ma et al., 2011), the nitrogen sources including organic or inorganic nitrogen sources in the fermentation medium can significantly affect the compositions of SL mixture. When yeast extract was used as the nitrogen source, a large amount of lactonic SLs was produced during the fermentation of the wild-type stain of *S. bombicola*. When ammonium sulfate was used as the nitrogen source, on the contrary, although the total SLs titer was the same as that of yeast extract, the synthesis of lactonic SLs was suppressed, and a large amount of acidic SLs is produced.

**FIGURE 1:**
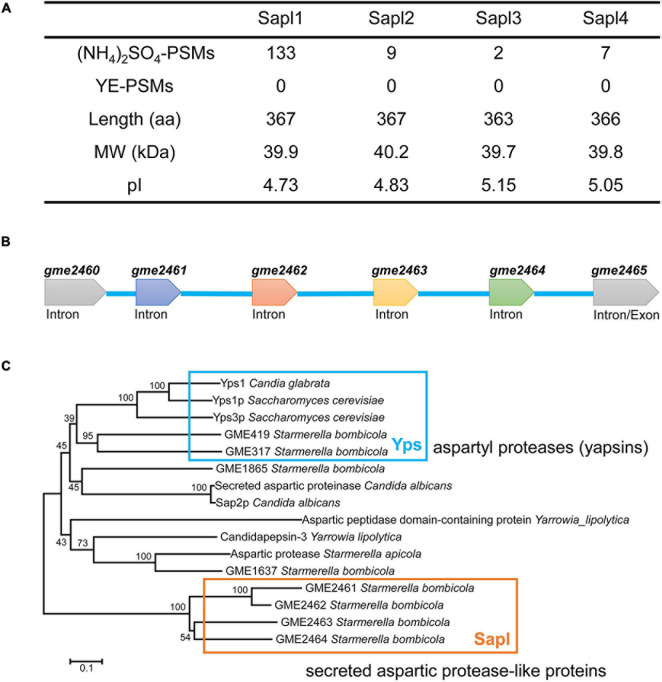
Bioinformation analysis of Sapl. **(A)** Primary extracellular protein with ammonium sulfate or yeast extract (YE) as source. **(B)** *sapl1*, *sapl2*, *sapl3*, and *sapl4* genes cluster. **(C)** Phylogenetic analysis of *S. bombicola* CGMCC 1576 Sapl1, Sapl2, Sapl3, and Sapl4 with their homologous proteins using the neighbor-joining (NJ) method.

By analyzing the proteomic data of the fermentation broth of *S. bombicola* under different nitrogen source conditions (yeast extract and ammonium sulfate), 78 extracellular proteins were found in the fermentation broth with yeast extract as the nitrogen source, and 153 extracellular proteins were found with ammonium sulfate as the nitrogen source (Supplementary Tables 2, 3). Differentially secreted proteins were enriched. Among them, GME2461 protein was the most secreted in the presence of ammonium sulfate and was not detected under the condition of yeast extract. The amino acid sequence of GME2461 is locally compared and analyzed with the whole genomic data, and four aspartic protease-like proteins were found. The four proteins were detected in the presence of only ammonium sulfate, but not found in the fermentation with yeast extract as the nitrogen source (as shown in Figure 1A). The coding genes of these four proteins are *gme2461*, *gme2462*, *gme2463*, and *gme2464* (Supplementary Material). The four genes are lined up in sequence on the same chromosome to form a gene cluster (Figure 1B). Among them, the protein encoded by *gme2461* is the most abundant protein. We conducted online comparison analysis and found that they contain a secreted aspartic protease (SAP)-like protein, which may be homologous to the Ysp1 protein of *Saccharomyces cerevisiae*, and the identity of the amino sequence with the Ysp1 protein of *S. cerevisiae* was 27.9, 27.7, 30.1, and 28.4%, respectively. However, when the Ysp1 protein sequence was subjected to local genome of *S. bombicola* blast analysis, the proteins GME317 and GME419 were detected and had the highest confidence, and the identity of the amino sequence was 34 and 31% with the Ysp1 protein of *S. cerevisiae*. Therefore, we performed a phylogenetic analysis of GME2461, GME2462, GME2463, and GME2464, and they are most closely related proteases in *S. bombicola* using the best hits of related yeast along with putative homologs from more distantly related yeast (Figure 1C). Accordingly, bidirectional best hit analysis showed that these four proteins were not conservative in yeast, not even in closely related *Starmerella apicola* and *Candida apicola*. The four proteins shared 27% identity of amino acid sequence with the secreted aspartic proteases 2 (Sap2) of *Candida albicans*. The structure of Sap2 of *C. albicans* has been resolved; thus, Sap2 was used for sequence alignment analysis. Consequently, GME2461, GME2462, GME2463, and GME2464 were named as SAP-like protein 1, 2, 3, and 4 (Sapl1, Sapl2, Sapl3, and Sapl4). Among the four proteins, Sapl1 and Sapl2 have higher sequence similarity, and the identity of the two amino acid sequences is as high as 82%. The sequence identity of Sapl1 and the other two proteins Sapl3 and Sapl4 is 55 and 59%, respectively. Sapl3 and Sapl4 have higher sequence identity, and the value is 62%.

**FIGURE 2:**
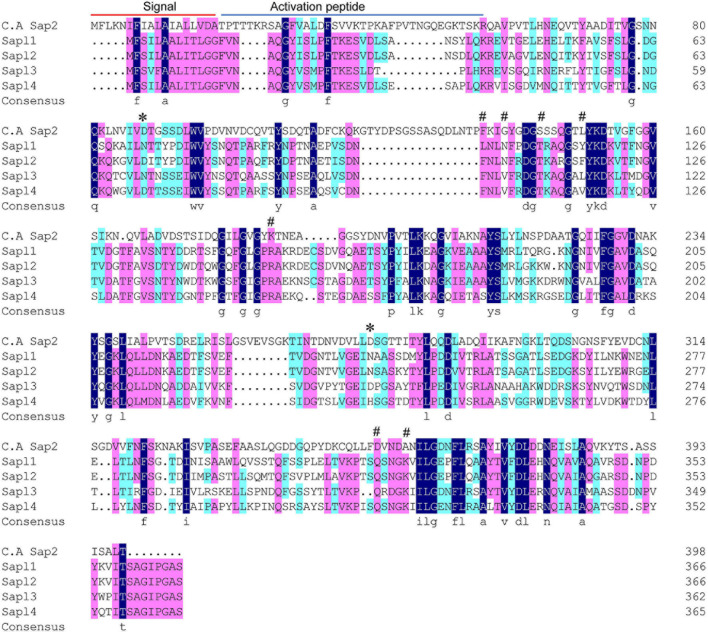
Protein sequence alignments of Sap2 (*C. albicans*), Sapl1, Sapl2, Sapl3, and Sapl4 (*S. bombicola*). The blocks in dark blue indicated the conserved domains, the residues marked with * represented the active sites, and the residues marked with # represented the inhibitor binding sites.

Multiple sequence alignment analysis on the amino acid sequences of the four proteins (*S. bombicola*) and Sap2 (*C. albicans*) was also performed, and the results are shown in Figure 2. There was a high degree of conservation among the four protein sequences, with multiple protein conserved regions. Moreover, the two active sites aspartic acid of Sap2 have been partially or completely mutated to asparagine, and it is likely that they have lost their activity.

### Sapl Negatively Impacts Key Gene Expression for SLs Synthesis

**FIGURE 3:**
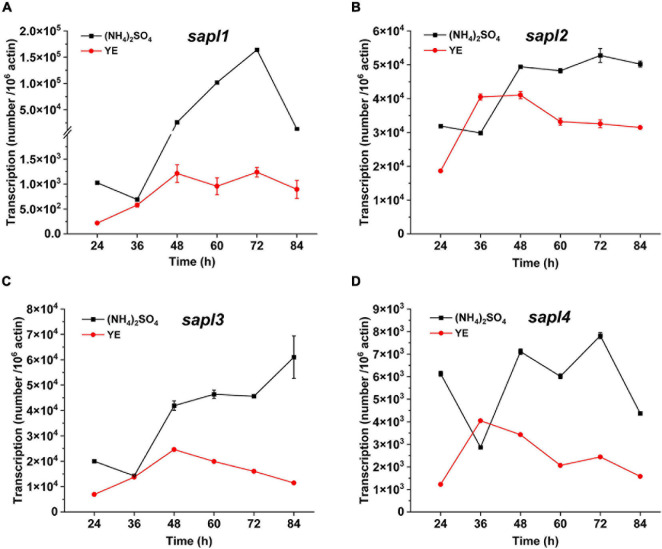
The different transcriptional levels of *sapl1*, *sapl2*, *sapl3*, and *sapl4* genes cultured in fermentation medium with different nitrogen sources. Ammonium sulfate and yeast extract (YE). **(A)** The trend of *sapl1* transcription level over time. **(B)** The trend of *sapl2* transcription level over time. **(C)** The trend of *sapl3* transcription level over time. **(D)** The trend of *sapl4* transcription level over time.

The expression levels of *sapl* genes under different nitrogen sources were determined by qRT-PCR. The results are shown in Figure 3. When ammonium sulfate was used as the nitrogen source, the transcription levels of the four genes were significantly upregulated compared with those in the case of yeast extract as the nitrogen source, especially the expression level of *sapl1* was upregulated 132 times at 72 h in the case of ammonium sulfate compared with that with yeast extract as the nitrogen source. The gene expression was also consistent with the proteomic results of the fermentation broth (Supplementary Tables 2, 3). Under the condition of ammonium sulfate, the protein content of Sapl1 was significantly higher than that in the case of yeast extract. These results indicated that the expression of *sapl* was induced by ammonium sulfate.

**FIGURE 4:**
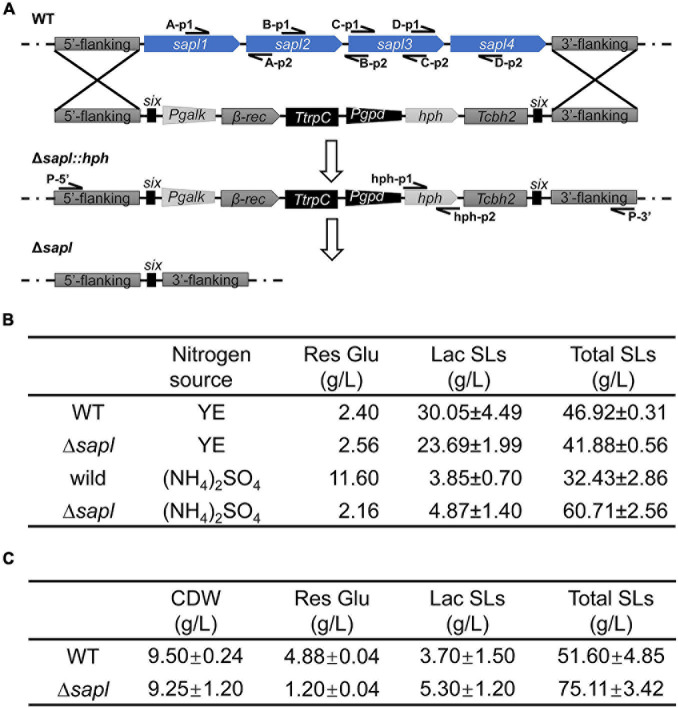
The effect of deletion of *sapl* genes on the detection of SLs. **(A)** Construction of *sapl* genes knockout mutant Δ*sapl*. Schematic illustration of the construction of Δ*sapl* mutant by homologous recombination, and the deletion of self-excising *hph* marker performed by expression of β-rec recombinase under the induction of galactose. **(B)** Fermentation profiles of the wild-type strain and Δ*sapl* strain for 5 days (WT, wild-type strain; YE, yeast extract; Res Glu, residual glucose; Lac SLs, lactone SLs; Total SLs, total SLs). **(C)** Fermentation profiles of the wild-type strain and Δ*sapl* strain for 7 days with ammonium sulfate as source (WT, wild-type strain; CDW, dry cell weight; Res Glu, residual glucose; Lac SLs, lactone SLs; Total SLs, total SLs).

In the genome of *S. bombicola*, four Sapl genes *sapl1*, *sapl2*, *sapl3*, and *sapl4* are included in a gene cluster, and the amino acid sequences of the four proteins have a high degree of homology. To study the function of these extracellular acidic proteins, a co-knockout strain of four *sapl* genes was constructed using hygromycin self-deletion selection markers (the specific strategies are shown in Figure 4A). When using primers (A-p1/A-p2, B-p1/B-p2, C-p1/C-p2, and D-p1/D-p2) for amplification, no specific bands appeared in the electrophoresis compared with the wild-type strain (Supplementary Figure 1), indicating that the four genes *sapl1*, *sapl2*, *sapl3*, and *sapl4* have been successfully knocked out.

**FIGURE 5:**
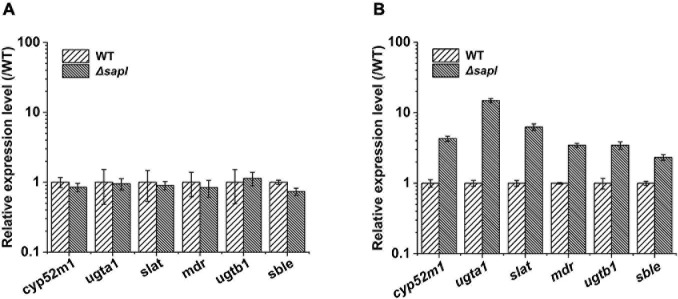
The different transcriptional levels of the key genes for SLs synthesis. **(A)** The yeast extract used as nitrogen source (WT, wild-type strain). **(B)** The ammonium sulfate used as nitrogen source (WT, wild-type strain).

In order to further explain the effect of *sapl* on the synthesis of SLs from *S. bombicola*, we explored the expression level of several key genes responsible for SLs synthesis in Δ*sapl* and wild-type strains with yeast extract and ammonium sulfate as the nitrogen source, respectively. Δ*sapl* and wild-type strains were cultivated in fermentation medium in triplicate. Samples for RNA extraction were taken after 72 h using the fluorescence quantitative polymerase chain reaction (qRT-PCR) detection (Figure 5).

The expression levels of four *sapl* genes were 1.6 × 10^5^, 5.3 × 10^4^, 1.6 × 10^4^, and 7.8 × 10^3^ in yeast extract (Figure 3); when ammonium sulfate was used as the nitrogen source, the expression of *sapl* genes was significantly induced. The expression level of the key genes for SLs synthesis in the wild-type strain under the condition of yeast extract was 1.5–3 times of that of key genes under the condition of ammonium sulfate. The expression levels of the key genes for SLs synthesis in Δ*sapl* were almost the same as those of the wild-type strain with yeast extract as the nitrogen source (Figure 5A). However, under the condition of ammonium sulfate, the expression level of key genes for SLs synthesis in Δ*sapl* was significantly upregulated compared with wild-type strains with ammonium sulfate as the nitrogen source, which was 1.7–9 times higher than that with yeast extract (Figure 5B).

Generally, long-chain fatty acids were catalyzed by Cyp52m1 to form hydroxy fatty acids (Van Bogaert et al., 2009). When ammonium sulfate was used as the nitrogen source, the expression level of *cyp52m1* in Δ*sapl* was 4.3 times higher than that of the wild-type strain. Subsequently, under the catalysis of Ugta1 and Ugtb1, two molecules of UDPG were linked to the fatty acid terminal or terminal hydroxyl group in the form of a special glycosidic bond, thereby forming a long-chain open-chain acidic SLs (Saerens et al., 2011a,c). The expression levels of *ugta1* and *ugtb1* were upregulated by 14.8 and 3.4 times, respectively. This may be the reason why the glucose conversion rate in the Δ*sapl* strain was higher than that in the wild-type strain. The acidic SLs were further modified by acetylation by Slat (Saerens et al., 2011b). Finally, acidic SLs were transported outside the cell by the transporter MDR (Van Bogaert et al., 2013). Similarly, the expression levels of *slat* and *mdr* were upregulated by 6.3 and 3.5 times, respectively. Therefore, the SLs production of Δ*sapl* strains increased significantly under the condition of ammonium sulfate as nitrogen source. *S. bombicola* lactone esterase (Sble) catalyzes the formation of acetylated acidic SLs into the lactonic SLs (Ciesielska et al., 2016). The expression of *sble* was upregulated by 2.3 times higher than that of the wild-type strain, which may be due to the inability to synthesize large amounts of lactonic SLs under (NH_4_)_2_SO_4_ conditions, and the expression of *sble* was increased by feedback regulation. However, the synthesized and secreted Sble protein may be low. In addition, the synthesis of lactonic SLs was affected by other esterase genes, so the upregulation of *sble* expression was not enough for the knockout strain to synthesize a large amount of lactonic SLs with ammonium sulfate as the nitrogen source. In summary, the absence of four *sapl* genes positively impacts SLs synthesis-related genes expression and also promoted the synthesis of acidic SLs in the Δ*sapl* strain.

### Enhancement of SLs Concentration in the Δ*sapl* Strain With Ammonium Sulfate as Nitrogen Source

In order to analyze the function of the Sapl protein and role of protein in SLs synthesis, the protein Sapl1 encoded by the *sapl1* gene with the highest expression level among the four *sapl* genes under ammonium sulfate conditions was heterologously expressed and purified in *P. pastoris* GS115. Sapl1 was used to determine acidic and neutral protease activities, using the casein method. The SDS-PAGE verification of the purified Sapl1 protein is shown in Supplementary Figure 2. The concentration of the Sapl1 protein was determined by Bradford to be 0.30 mg/ml, but no protease activity was detected; the reason for which may be that the mutation of the aspartic acid in the active site of Sapl1 to asparagine leads to the inactivity of the protease activity of Sapl1 protein.

In order to investigate the role that Sapl protein played in the synthesis of SLs from *S. bombicola*, Δ*sapl* and wild-type strains were grown in fermentation medium with yeast extract or ammonium sulfate as nitrogen sources for 5 days at 30°C at 200 rpm. The titer of SLs and the amount of residual glucose were determined (Figure 4B). When yeast extract was used as the nitrogen source, compared with the wild-type strain, the total SLs and lactonic SLs in Δ*sapl* both decreased slightly. The titer of the total SLs of Δ*sapl* decreased from 46.92 to 41.88 g/L, and the content of lactonic SLs decreased from 30.05 to 23.69 g/L. When ammonium sulfate was used as the nitrogen source, the titer of SLs by Δ*sapl* increased significantly compared with that by the wild-type strain, the total SLs reached 60.71 g/L, and the concentration of acidic SLs was 55.84 g/L. The proportion of acidic SLs produced by the Δ*sapl* strain accounted for 92% in the total SLs. The residual glucose was 2.16 g/L after 5 days of cultivation of Δ*sapl*, resulting in a productivity of 12 g L^–1^ d^–1^, while glucose of the wild-type strain remained at 11.60 g/L, and the productivity was only 6 g L^–1^ d^–1^. An overall consumption of 77.84 g/L glucose led to a yield coefficient of Y_*SL/Glu*_ = 0.78 (g SL formed/g glucose consumed) in Δ*sapl*, while the Y_*SL/Glu*_ was only 0.47 in the wild-type strain. The results indicated that Δ*sapl* has a more efficient glucose utilization rate than the wild-type strain. The knockout strain not only increased the production of acidic SLs but also enhanced glucose conversion.

Fermentation time of Δ*sapl* and wild-type strains using ammonium sulfate as nitrogen source was prolonged to 7 days (Figure 4C). The titer of total SLs of Δ*sapl* increased from 60.71 to 75.11 g/L, and acidic SLs were the main contributors for the total SLs. With the fermentation period from 5 days to 7 days, the total SLs titer of the wild-type strain also increased to 51.6 g/L and showed a higher volumetric productivity within the last 2 days of cultivation. These results indicated that glucose consumption and SLs synthesis were retarded in the wild-type strain compared with the deletion strain Δ*sapl*. The time-dependent graph of SLs synthesis under ammonium sulfate conditions is shown in Supplementary Figure 3. In the early stage, the synthesis of SLs in Δ*sapl* was basically the same as that of the wild-type strain. After the third day, Δ*sapl* began to synthesize in a large amount, and the wild-type strain still proceeded at a slower speed (the slope of the curve was lower). Therefore, SLs synthesis in Δ*sapl* was faster than that in wild-type strains under ammonium sulfate conditions. In the later stage, the synthesis rate of SLs in Δ*sapl* decreased because of the insufficient supply of carbon sources in the medium. From the above results, the performance of the knockout strains was markedly different when fermented with different nitrogen sources. Under the condition of yeast extract, the SLs titer of Δ*sapl* was slightly reduced, while under the condition of ammonium sulfate, the total and acidic SLs titer of Δ*sapl* increased by 90 and 100%, compared with that of the wild-type strains, respectively. Thus, we wondered whether the expression of key enzyme genes for SLs synthesis was affected by the Sapl protein.

### Strain Growth and Glucose Consumption With Yeast Extract or Ammonium Sulfate as Nitrogen Source

**FIGURE 6:**
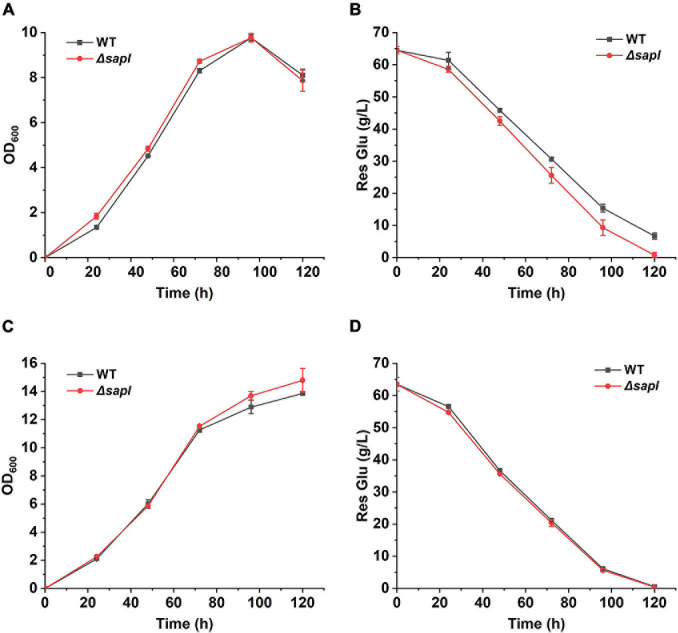
The growth assay of wild-type and Δ*sapl* with different nitrogen sources. **(A,B)** Measure the absorbance of OD_600_ and residual glucose with ammonium sulfate as nitrogen source. **(C,D)** Measure the absorbance of OD_600_ and residual glucose with yeast extract as nitrogen source.

In order to further explore whether the lack of *sapl* improves the growth ability and SLs production of the Δ*sapl* strain with ammonium sulfate as the nitrogen source in Δ*sapl*, the growth of Δ*sapl* and wild-type strains was analyzed under two different nitrogen sources. As shown in Figure 6, under the conditions of fermentation with yeast extract or ammonium sulfate as the nitrogen source, the growth of Δ*sapl* was almost the same as that of the wild-type strain. The glucose consumption rate in Δ*sapl* was equivalent to that of the wild-type strain with yeast extract as the nitrogen source. However, under the condition of ammonium sulfate, the glucose consumption rate was slightly faster than that of the wild-type strain. This was consistent with the result that the SLs production in Δ*sapl* was higher than that in the wild-type strain with ammonium sulfate as the nitrogen source.

## Discussion

The non-pathogenic yeast *S. bombicola* has been known for decades as an outstanding producer of SLs. Naturally synthesized SLs were a mixture of dozens of SL molecules, mainly divided into two categories, namely, acidic and lactonic SLs. Lactonic SLs had better antibacterial and anti-tumor biological activities, while acidic SLs can be widely used in the repair of ecological environments and used as surfactants.

In order to reduce the purification cost of SLs, 106 mutants from 7 batches were screened by atmospheric and room temperature plasma for improved production of specific or total SLs. Mutants of A2-8 performed comparable acidic and total SLs production of 68.75 and 100.33 g/L in fed-batch cultivation, and the proportion of acidic SLs reached 68.52% in the total SLs (Ma et al., 2020). Ciesielska et al. (2014) found that the esterase (Sble) was required for SLs lactonization in *S. bombicola* by analyzing exoproteome. The deletion (as opposed to overexpression) of the lactone esterase gene clearly also has a very pronounced effect on SL production, as no lactonic SLs (as no acidic SLs) were detected in the fermentation broth of the mutant strain. Roelants et al. (2016) used the knockout strain of the lactone esterase gene to carry out feed fermentation in 3-L fermenter for 6 days, the knockout strain produced 124 g/L acidic SLs, and the value of the carbon source conversion efficiency yield (g/g) was 0.41 [yield (g/g) represents the ratio of the yield of SLs to the total amount of added substrate (glucose + oil)].

In this study, a cluster including four Sapl protein-encoding genes was identified. Sequence alignment analysis revealed that one or two active sites of the two aspartic acids were mutated to asparagine, so the Sapl protein was likely to have no protease activity and the speculation was supported by the absence of protease activity. It was possible that the binding domain of the Sapl protein can still play a role and continue to perform its functions, thereby affecting the growth and metabolism of the strain under the condition of ammonium sulfate as a nitrogen source. Interestingly, after the four *sapl* genes were knocked out together, the productivity of SLs production in Δ*sapl* reached 12 g L^–1^ d^–1^, which was twofold of that of the wild-type strain with ammonium sulfate as the nitrogen source, and the total SLs production in Δ*sapl* increased by 90% compared with the wild-type strain, and acidic SLs accounted for the vast majority, with a proportion up to 92%. Under the condition of ammonium sulfate, the expression of the four *sapl* genes was significantly induced, and the expression of the key genes involved in SLs synthesis was enhanced after deletion of the four *sapl* genes, which significantly increased the titer of SLs in Δ*sapl*. With yeast extract as a nitrogen source, the expression of the four *sapl* genes was inhibited, and the deletion of the four *sapl* genes had no effect on the expression of the key genes for SLs synthesis and the SLs production in Δ*sapl*. In *S. bombicola*, glucose consumption mainly occurs in growth metabolism and SLs synthesis pathways (the UDPG, which was the substrate for SLs synthesis, was derived from glucose metabolism). Under the condition of ammonium sulfate, the glucose consumption rate in Δ*sapl* was slightly faster than that in the wild-type strain, and the growth was almost the same as that of the wild-type strain, which indicated that the deletion of the *sapl* gene promoted the glucose metabolism pathway for SLs synthesis in Δ*sapl*. The expression levels of *ugta1* and *ugtb1* in Δ*sapl* were upregulated by 14.8 and 3.4 times compared with the wild-type strain, respectively.

The synthesis of SLs is regulated by the telomere positioning effect. During the exponential growth phase, the synthesis pathway is strictly regulated, and SLs cannot be synthesized at this time. When the second copy of the SLs biosynthesis gene cluster was introduced into the genome far away from the telomeres, the mutant strain began to synthesize SLs during the exponential phase (Lodens et al., 2020). Secretion of aspartic protease is attached to the plasma membrane and participates in the cell wall integrity response in *S. cerevisiae* (Krysan et al., 2005). The four Sapl proteins have not been reported to affect the synthesis of SLs in *S. bombicola*. This is the first time to reveal how SLs synthesis was influenced under the condition of ammonium sulfate. The four Sapl proteins can be considered as potential regulatory proteins for SLs synthesis under ammonium salt conditions.

*Candida batistae* has been identified as mainly producing acidic SLs (more than 60% of the total SLs), although the total yield is low, 6 g/L (Konishi et al., 2008). In the fed-batch fermentation of *C. batistae* using a 50 L fermentor, the SLs production reached 53.2 g/L, and productivity was 8.87 g L^–1^ d^–1^ (Kim et al., 2021). Unlike *S. bombicola*, the SLs synthesized by *Rhodotorula bogoriensis* contain a docosanoic acid (C22 fatty acid) instead of C16 or C18 fatty acid and only produce acidic SLs. SLs yield is 1.26 g/L with rapeseed oil as a hydrophobic substrate (Zhang et al., 2011). The yield of acidic SLs was 55.84 g/L and increased productivity to 11.17 g L^–1^ d^–1^.

The unique performance of deletion strain Δ*sapl* can reduce the production and separation costs of SLs. At the same time, the upregulation of the expression levels of the key enzymes responsible for SLs synthesis in the deleted strains of these four genes provides new idea for the construction of high-yield and high-production acidic SLs-producing strains by further overexpressing these key enzymes.

## Data Availability Statement

The datasets presented in this study can be found in online repositories. The names of the repository/repositories and accession number(s) can be found in the article/Supplementary Material.

## Author Contributions

XS and JL conceived and designed the research. JL, GZ, and XZ conducted the experiments. JL and GZ analyzed the data. JL wrote the manuscript. All authors read and approved the manuscript.

## Conflict of Interest

The authors declare that the research was conducted in the absence of any commercial or financial relationships that could be construed as a potential conflict of interest.

## Publisher’s Note

All claims expressed in this article are solely those of the authors and do not necessarily represent those of their affiliated organizations, or those of the publisher, the editors and the reviewers. Any product that may be evaluated in this article, or claim that may be made by its manufacturer, is not guaranteed or endorsed by the publisher.

## Footnotes

1
http://blast.ncbi.nlm.nih.gov/Blast.cgi

## References

[B1] Andersen K. K. Vad B. S. Roelants S. van Bogaert I. N. Otzen D. E. (2016). Weak and Saturable Protein-Surfactant Interactions in the Denaturation of Apo-alpha-Lactalbumin by Acidic and Lactonic Sophorolipid. *Front. Microbiol.* 7:1711. 10.3389/fmicb.2016.01711 27877155 PMC5099233

[B2] Anjum F. Gautam G. Edgard G. Negi S. (2016). Biosurfactant production through *Bacillus* sp MTCC 5877 and its multifarious applications in food industry. *Bioresour. Technol.* 213 262–269. 10.1016/j.biortech.2016.02.091 27013189

[B3] Buschmann N. Wodarczak S. (1995). Analytical methods for alkylpolyglucosides. Part I: colorimetric determination. *Tenside Surfact. Det.* 32 336–339. 10.1002/lipi.19960981205

[B4] Ciesielska K. Roelants S. L. Van Bogaert I. N. De Waele S. Vandenberghe I. Groeneboer S. (2016). Characterization of a novel enzyme-*Starmerella bombicola* lactone esterase (SBLE)-responsible for sophorolipid lactonization. *Appl. Microbiol. Biotechnol.* 100 9529–9541. 10.1007/s00253-016-7633-2 27251547

[B5] Ciesielska K. Van Bogaert I. N. Chevineau S. Li B. Groeneboer S. Soetaert W. (2014). Exoproteome analysis of *Starmerella bombicola* results in the discovery of an esterase required for lactonization of sophorolipids. *J. Proteomics* 98 159–174. 10.1016/j.jprot.2013.12.026 24418522

[B6] Develter D. W. G. Lauryssen L. M. L. (2010). Properties and industrial applications of sophorolipids. *Eur. J. Lipid Sci. Tech.* 112 628–638. 10.1002/ejlt.200900153

[B7] Gaur V. K. Regar R. K. Dhiman N. Gautam K. Srivastava J. K. Patnaik S. (2019). Biosynthesis and characterization of sophorolipid biosurfactant by *Candida* spp.: application as food emulsifier and antibacterial agent. *Bioresour. Technol.* 285:121314. 10.1016/j.biortech.2019.121314 30992159

[B8] Griffin N. M. Yu J. Y. Long F. Oh P. Shore S. Li Y. (2010). Label-free, normalized quantification of complex mass spectrometry data for proteomic analysis. *Nat. Biotechnol.* 28 83–89. 10.1038/nbt.1592 20010810 PMC2805705

[B9] Hirata Y. Ryu M. Oda Y. Igarashi K. Nagatsuka A. Furuta T. (2009). Novel characteristics of sophorolipids, yeast glycolipid biosurfactants, as biodegradable low-foaming surfactants. *J. Biosci. Bioeng.* 108 142–146. 10.1016/j.jbiosc.2009.03.012 19619862

[B10] Kim J. H. Oh Y. R. Han S. W. Jang Y. A. Hong S. H. Ahn J. H. (2021). Enhancement of sophorolipids production in *Candida batistae*, an unexplored sophorolipids producer, by fed-batch fermentation. *Bioprocess Biosyst. Eng.* 44 831–839. 10.1007/s00449-020-02493-4 33683450

[B11] Konishi M. Fukuoka T. Morita T. Imura T. Kitamoto D. (2008). Production of New Types of Sophorolipids by *Candida batistae*. *J. Oleo Sci.* 57 359–369. 10.5650/jos.57.359 18469499

[B12] Krysan D. J. Ting E. L. Abeijon C. Kroos L. Fuller R. S. (2005). Yapsins are a family of aspartyl proteases required for cell wall integrity in *Saccharomyces cerevisiae*. *Eukaryot. Cell* 4 1364–1374. 10.1128/Ec.4.8.1364-1374.2005 16087741 PMC1214537

[B13] Li J. Li H. Li W. Xia C. Song X. (2016a). Identification and characterization of a flavin-containing monooxygenase MoA and its function in a specific sophorolipid molecule metabolism in *Starmerella bombicola*. *Appl. Microbiol. Biotechnol.* 100 1307–1318. 10.1007/s00253-015-7091-2 26512005

[B14] Li J. Xia C. Fang X. Xue H. Song X. (2016b). Identification and characterization of a long-chain fatty acid transporter in the sophorolipid-producing strain *Starmerella bombicola*. *Appl. Microbiol. Biotechnol.* 100 7137–7150. 10.1007/s00253-016-7580-y 27183996

[B15] Liu J. Li J. Gao N. Zhang X. Zhao G. Song X. (2020). Identification and characterization of a protein Bro1 essential for sophorolipids synthesis in *Starmerella bombicola*. *J. Ind. Microbiol. Biotechnol.* 47 437–448. 10.1007/s10295-020-02272-w 32377991

[B16] Lodens S. Roelants S. Luyten G. Geys R. Coussement P. De Maeseneire S. L. (2020). Unraveling the regulation of sophorolipid biosynthesis in *Starmerella bombicola*. *FEMS Yeast Res.* 20:foaa021. 10.1093/femsyr/foaa021 32329773

[B17] Ma X. J. Li H. Shao L. J. Shen J. Song X. (2011). Effects of nitrogen sources on production and composition of sophorolipids by *Wickerhamiella domercqiae* var. sophorolipid CGMCC 1576. *Appl. Microbiol. Biotechnol.* 91 1623–1632. 10.1007/s00253-011-3327-y 21590287

[B18] Ma X. J. Zhang H. M. Lu X. F. Han J. Zhu H. X. Wang H. (2020). Mutant breeding of *Starmerella bombicola* by atmospheric and room-temperature plasma (ARTP) for improved production of specific or total sophorolipids. *Bioprocess Biosyst. Eng.* 43 1869–1883. 10.1007/s00449-020-02377-7 32447514

[B19] Qi X. Y. Xu X. M. Zhong C. Q. Jiang T. Y. Wei W. Song X. (2018). Removal of Cadmium and Lead from Contaminated Soils Using Sophorolipids from Fermentation Culture of *Starmerella bombicola* CGMCC 1576 Fermentation. *Int. J. Environ. Res. Public Health* 15:2334. 10.3390/ijerph15112334 30360495 PMC6267470

[B20] Roelants S. L. Ciesielska K. De Maeseneire S. L. Moens H. Everaert B. Verweire S. (2016). Towards the industrialization of new biosurfactants: biotechnological opportunities for the lactone esterase gene from *Starmerella bombicola*. *Biotechnol. Bioeng.* 113 550–559. 10.1002/bit.25815 26301720

[B21] Saerens K. M. J. Roelants S. L. K. W. Van Bogaert I. N. A. Soetaert W. (2011a). Identification of the UDP-glucosyltransferase gene UGTA1, responsible for the first glucosylation step in the sophorolipid biosynthetic pathway of *Candida bombicola* ATCC 22214. *FEMS Yeast Res.* 11 123–132. 10.1111/j.1567-1364.2010.00695.x 21073653

[B22] Saerens K. M. J. Saey L. Soetaert W. (2011b). One-Step Production of Unacetylated Sophorolipids by an Acetyltransferase Negative *Candida bombicola*. *Biotechnol. Bioeng.* 108 2923–2931. 10.1002/bit.23248 21702032

[B23] Saerens K. M. J. Zhang J. Saey L. Van Bogaert I. N. A. Soetaert W. (2011c). Cloning and functional characterization of the UDP-glucosyltransferase UgtB1 involved in sophorolipid production by *Candida bombicola* and creation of a glucolipid-producing yeast strain. *Yeast* 28 279–292. 10.1002/yea.1838 21456054

[B24] Santos D. K. Rufino R. D. Luna J. M. Santos V. A. Sarubbo L. A. (2016). Biosurfactants: multifunctional Biomolecules of the 21st Century. *Int. J. Mol. Sci.* 17:401. 10.3390/ijms17030401 26999123 PMC4813256

[B25] Singh P. Cameotra S. S. (2004). Potential applications of microbial surfactants in biomedical sciences. *Trends Biotechnol.* 22 142–146. 10.1016/j.tibtech.2004.01.010 15036865

[B26] Valotteau C. Baccile N. Humblot V. Roelants S. Soetaert W. Stevens C. V. (2019). Nanoscale antiadhesion properties of sophorolipid-coated surfaces against pathogenic bacteria. *Nanoscale Horiz.* 4 975–982. 10.1039/c9nh00006b

[B27] Van Bogaert I. N. Holvoet K. Roelants S. L. Li B. Lin Y. C. Van de Peer Y. (2013). The biosynthetic gene cluster for sophorolipids: a biotechnological interesting biosurfactant produced by *Starmerella bombicola*. *Mol. Microbiol.* 88 501–509. 10.1111/mmi.12200 23516968

[B28] Van Bogaert I. N. A. De Mey M. Develter D. Soetaert W. Vandamme E. J. (2009). Importance of the cytochrome P450 monooxygenase CYP52 family for the sophorolipid-producing yeast *Candida bombicola*. *FEMS Yeast Res.* 9 87–94. 10.1111/j.1567-1364.2008.00454.x 19054129

[B29] Van Bogaert I. N. A. Zhang J. Soetaert W. (2011). Microbial synthesis of sophorolipids. *Process Biochem.* 46 821–833. 10.1016/j.procbio.2011.01.010

[B30] Zhang J. X. Saerens K. M. J. Van Bogaert I. N. A. Soetaert W. (2011). Vegetable oil enhances sophorolipid production by *Rhodotorula bogoriensis*. *Biotechnol. Lett.* 33 2417–2423. 10.1007/s10529-011-0703-8 21769647

