# Supplementary material for: Identification of Four Secreted Aspartic Protease-Like Proteins Associated With Sophorolipids Synthesis in Starmerella bombicola CGMCC 1576
Source: Front Microbiol. 2021 Sep 14;12:737244. doi: 10.3389/fmicb.2021.737244 (PMC8476993; doi:10.3389/fmicb.2021.737244)
Supplement: Supplementary file 1 [file Data_Sheet_1.docx]

Supplementary Material

# Supplementary Data

**Sequence information**

*gme2461*: ATGTTCTCTATACTCGCAGCACTCATTACTCTTGGCGGTTTCGTCAATGCTCAAGGCTATATCTCTTTGCCTTTCACAAAGGAGAGTGTAGACCTTTCAGCGAACTCTTACCTTCAGAAGAGAGAAGTCACCGGAGAGCTCGAACATGAGCTCACCAAATTTGCCGTTAGCTTCTCTCTCGGCGATGGCCAGAGTCAGAAGGCTATTCTTAACACCACCTACCCAGACATTTGGGTTTACAGCAACCAGACGCCAGCTCGATTCAGGTACAACCCGACCAACGCCGAGCCTGTCAGTGACAACTTAAATTTGAATTTCCCCGACGGAACCAGGGCACAAGGTTCCTACTACAAGGACAAAGTGACTTTCAATGGTGTTACCGTTGATGGTACGTTCGCTGTTTCCAACACTTACGACGACAGAACTTCGTTCGGCCAGTTCGGACTCGGTCCCCGGGCCAAGAGAGACGAGTGCAGCGATGTAGGTCAGGCTGAAACTAGCTATCCTTATATCCTCAAGGAAGCGGGTAAAGTCGAGGCCGCGGCCTACTCTATGCGCTTGACTCAGAGAGGTAAAAATGGTAACATTGTCTTTGGTGCCGTTGATGCCTCTCAGTACGAAGGAAAGCTCCAGCTGCTCGACAACAAGGCTGAAGACACCTTCTCAGTAGAATTCACTGTTGATGGCAACACCCTCGTCGGTGAGATCAACGCAGCGTCTAGTGACATGTACCTGCCGGACGATATTGTCACTCGCCTTGCCACTTCTTCCGGTGCAACTCTGAGCGAAGACGGTAAAGATTACATTTTGAACAAATGGAATGAAAACTTGGAGTTGACTCTCAACTTCTCTGGCACGGATATCAACATCTCCGCTGCATGGCTACAGGTCTCTTCGACGCAGTTTAGTAGCCCGTTAGAGCTCACTGTGAAACCCACTTCTCAGAGCAATGGCAAGGTTATTCTCGGTGAGCCCTTCTTGCAAGCTGCATACACTGTATTCGATCTTGAACATAATCAGGTGGCAGTTGCTCAGGCAGTTAGAAGTGATAACCCAGATTATAAGGTCATTACTAGCGCTGGTATTCCTGGGGCTTCGTATTAA

*gme2462*:

ATGTTCTCTATACTCGCAGCACTTATTACCCTTGGCGGTTTCGTCAATGCTCAAGGCTATATCTCTTTGCCTTTCACAAAGGAGAGTGTAGACCTTTCAGCGAACTCTGACCTTCAGAAGAGAGAAGTCGCCGGAGTTCTTGAGAATCAGATCACCAAATATATCGTTAGCTTCTCTCTCGGCGATGGCCAGAAACAGAAGGGTGTTCTTGACATCACCTACCCAGACATTTGGGTTTACAGCAACCAGACGCCAGCTCAATTCAGGTACGACCCGACCAACGCCGAGACCATCAGTGACAACTTCAATCTGAATTTCCGCGACGGAACCAAGGCACGCGGTTCCTTCTACAAGGACAAAGTGACTTTCAATGGTGTTACCGTTGATGGTACGTTCGCTGTTTCCGACACTTACGACTGGGACACTCAGTGGGGCCAGTTTGGGCTCGGTCCCCGGGCCAAGAGAGACGAGTGCAGCGATGTAAATCAGGCTGAAACTAGCTATCCTTATATCCTCAAGGATGCGGGTAAAATCGAGGCCGCGGCCTACTCTATGCGCTTGGGTAAGAAATGGAAAAATGGTAACATTGTCTTTGGTGCCGTTGATGCCTCTCAGTACGAAGGAAAGCTCCAGCTGCTCGACAACAAGGCTGAAGACACCTTCTCAGTAGAATTCACTGTTGATGGCAACACCGTCGTCGGTGAGCTCAACTCAGCGTCTAAGTACACGTACCTGCCGGACGATGTTGTCACTCGCCTTGCCACTTCTGCCGGTGCAACTCTGAGCGAAGACGGTAAAAGTTACATTTTGTACGAATGGAGGGGAGAACTGGAGTTGACTCTCAACTTCTCCGGCACGGATATCATCATGCCCGCTAGCACGCTACTGAGCCAAATGACGCAGTTTTCTGTGCCGTTGATGCTCGCTGTGAAACCCACTTCTCAGAGCAATGGCAAGGTTATTCTCGGTGAGCCCTTCTTGCAAGCTGCATACACTGTATTCGATCTTGAACATAACCAGGTGGCAATTGCTCAGGGTGCCCGAAGTGATAACCCAGATTATAAGGTCATTACTAGCGCTGGTATTCCTGGGGCTTCGTATTAA

*gme2463*:

ATGTTTTCTGTATTCGCAGCACTTATTACTCTTGGCGGTTTCGTCAATGCTCAGGGCTATGTCTCTATGCCGTTCACGAAGGAAAGTTTGGACACACCTCTCCACAAGAGAGAAGTTTCCGGCCAAATTCGAAACGAGAGATTTCTCTATACAATTGGCTTCTCTCTTGGCAATGACCAGAAACAGACGTGTGTTCTCAACACGAACTCCTCAGAGATCTGGGTTTACAACTCCCAGACTCAAGCTGCATCCAGCTACAACCCGAGCGAGGCCCAGCTTGTCAGTGACAACTTTAACCTGGTTTTCCGCGATGGAACCAAGGCACAAGGTGCCCTCTACAAGGATAAACTGACTATGGATGGTGTTACTGTTGATGCCACGTTTGGCGTTTCCAATACTTACAATTGGGACACAAAGTGGGGCAGCTTCGGAATTGGCCCCCGGGCCGAGAAAAACAGTTGCAGCACGGCAGGTGATGCTGAAACTAGTTATCCTTTTGCCCTCAAGAATGCGGGTAAAATCGAGGCAGCGGCCTACTCTCTGGTCATGGGCAAGAAAGACCGCTGGAATGGTGTTGCTCTCTTCGGTGCCGTGGATGCCACTGCATACCAAGGAAAGCTTCAGCTGTTGGACAATCAGGCTGATGACGCCATCGTAGTAAAATTCTCTGTTGATGGCGTTCCATACACCGGCGAGATAGACCCAGGTTCTGCTTATACGTTCCTCCCGGAAGATATCGTCGGTCGCCTCGCTAATGCTGCCCATGCAAAGTGGGATGATCGATCCAAGTCTTACAACGTTCAAACATGGTCTGACAATCTAACCCTGACCATCCGGTTCGGCGACATCGAAATTGTTCTTCGCTCTAAGGAGCTGCTGAGCCCGAACGATCAGTTTGGGTCGTCCTACACGCTAACTGTGAAGCCCCAGAGAGACGGTAAGATCATTCTCGGTGACAACTTCTTGCGATCTGCCTATACAGTTTATGATCTTGAGCGTAACCAGGTGGCAATTGCTATGGCTGCCTCGAGTGATGATAACCCTGTTTATTGGCCTATCACCAGCGCTGGTATTCCTGGGGCTTCGTATTAA

*gme2464*:

ATGTTCTCTATACTCGCAGCTCTTATTACTCTTGGCGGTTTCGTCAATGCTCAAGGCTATGTCTCTATGCCATTCACGAAAGAGAGTGTGGACCTCTCGGAGAGCGCACCTCTCCAGAAGAGAGTAATTTCCGGAGATGTCATGAATCAGATCACCACATATACCGTTGGCTTCACTCTTGGCAACGGCCAGAAACAGTGGGGTGTTCTCGACACGACCTCCTCAGAGATCTGGGTTTACAGCTCTCAGACTCCAGCTCGGTTCAGCTACAACCCGAGCGAGGCCCAGTCTGTCTGTGACAACTTCAATCTGGTTTTCCGCGATGGAACCAAGGCTCAAGGTGCCGTCTACAAGGACAAACTGACTTATCAGGATGTTAGTCTTGATGCTACGTTTGGTGTCTCCAATACTTACGATGGGAATACTCCTTTCGGCACTTTCGGAATTGGCCCCCGGGCTGAGAAACAAAGCACGGAAGGTGATGCTGAAAGCAGTTTTCCTTATGCTCTCAAGAAGGCGGGTCAAATCGAGACCGCTTCCTATTCTTTGAAAATGAGCAAGAGAGGCTCGGAAGACGGTCTCATTACCTTCGGTGCCCTGGACAGGAAGTCATACGTAGGAAAGCTTCAGCTAATGGATAATCTGGCTGAGGATGTCTTCAAGGTGAATTTCAGTATTGATGGCACTTCACTCGTTGGCGAGATCCACTCAGGGTCTACCGATACGTACCTACCGGATGATATCGTCTCTCGCCTCGCTGCTTCTGTCGGTGGAAGGTGGGATGAAGTGAGTAAGACTTACCTTGTAGATAAGTGGACTGACTATCTGCTCCTGTACCTCAACTTTTCCGACACCTATATTGCGATTCCCGCTCCTTACCTGCTGAAGCCCATCAATCAGTCTCGTTCGGCCTACTCGCTAACTGTGAAACCCATTTCTCAGAGCAACGGTAAGATCATTCTCGGTGAAAACTTCTTACGCGCTGCCTTAACTGTGTATGATCTTGAGCGTAACCAGATTGCCATTGCTCAGGCTACTGGGAGCGATTCCCCTTACTACCAGACTATTACTAGCGCTGGTATTCCTGGGGCTTCGTATTAA

# Supplementary Figures and Tables

## Supplementary Figures
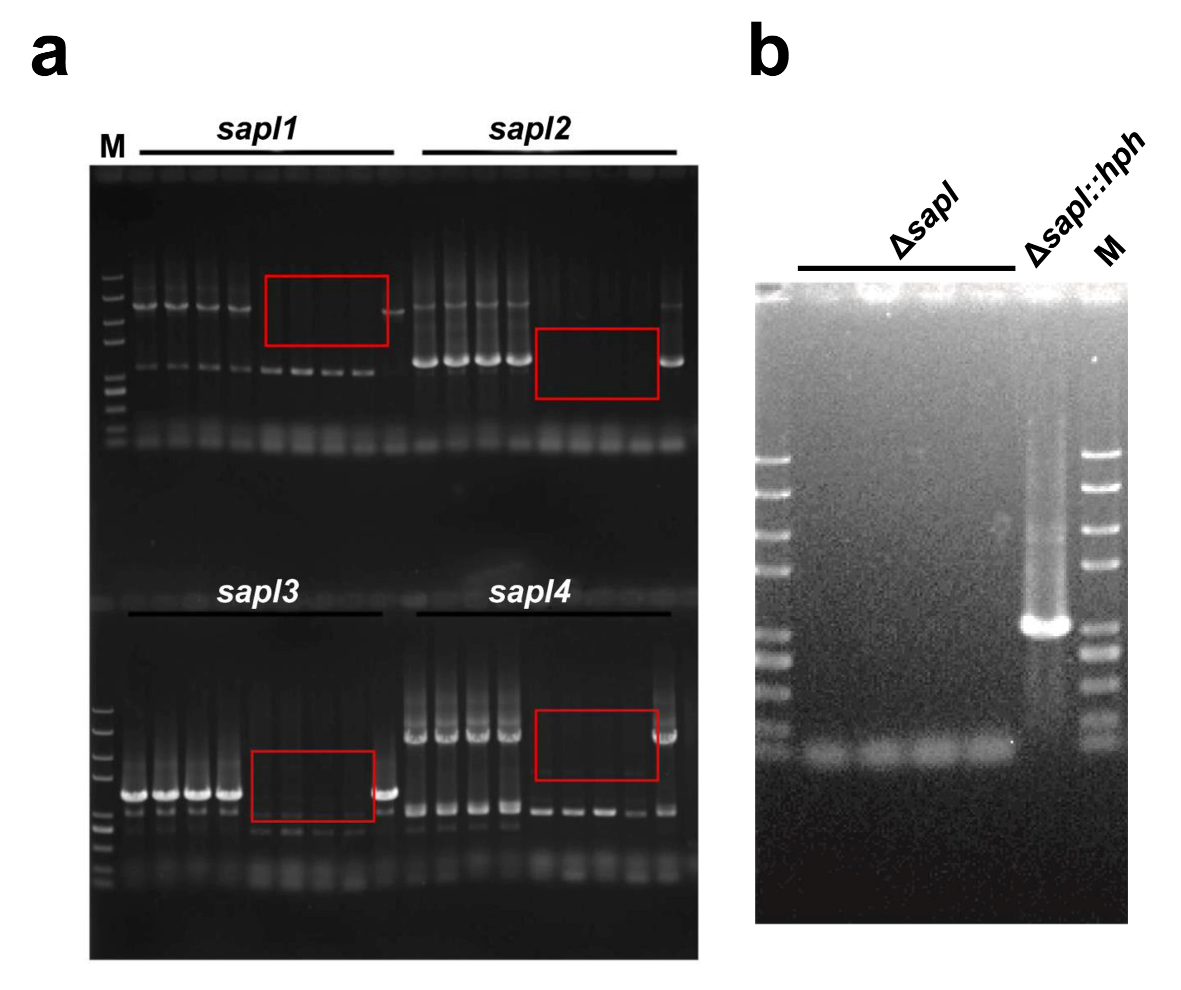


**Supplementary Figure 1.** Identification of the deletion mutants by PCR amplification. **(a)** PCR amplification using the primers within the target genes *sapl1*, *sapl2*, *sapl3* and *sapl4*, A-p1/A-p2, B-p1/B-p2, C-p1/C-p2 and D-p1/D-p2, the verified mutants of Δ*sapl* were marked by red boxes. **(b)** Identification by PCR using primers within *hph* coding sequence, hph-p1 and hph-p2.


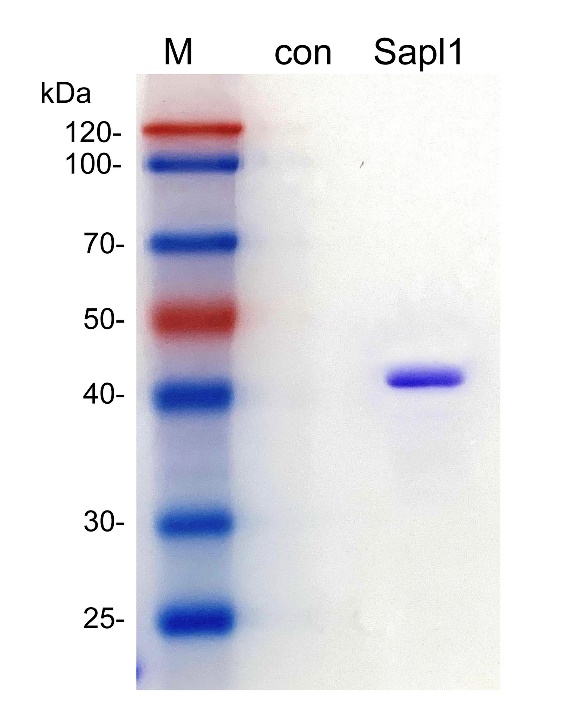


**Supplementary Figure 2.** SDS-PAGE analysis of the Sapl1 protein. *con*: Empty pPIC9K plasmid control.


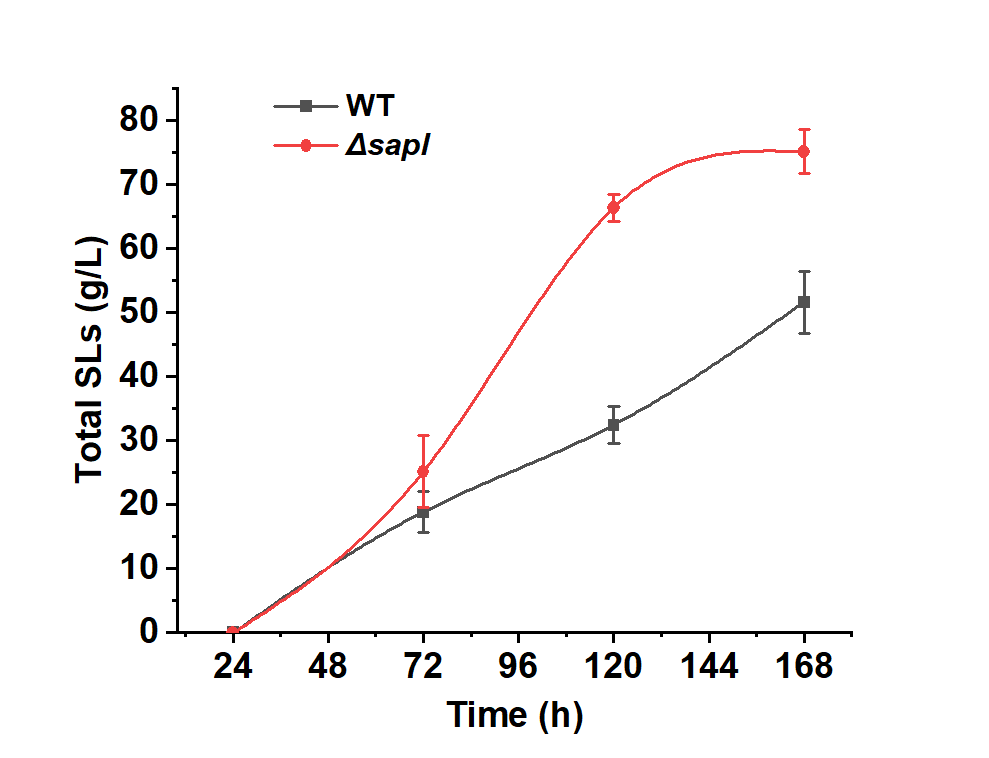


**Supplementary Figure 3.** The total SLs synthesis under ammonium sulfate conditions.

## Supplementary Tables

**Supplementary Table S1.** Primers used for this study

| Group | Primers | Sequence (5’ to 3’) |
| --- | --- | --- |
| **Primers used in the construction of deletion mutants** | | |
| ∆*sapl* | 5’flankingfor | AGCACTTCCATTAGCAATCTCG |
|  | 5’flankingrev | CTGATGCGGTATTTTCTCCTTACGCCAAATTGTAGGGAACGAACGAGT |
|  | 3’flankingfor | AACATACGAGCCGGAAGCATAAAGTAGACTTTTCTAGCCCATTCGTGA |
|  | 3’flankingrev | AGCTTCATGAGAAACCGCACA |
|  | rec-hphfor | GCGTAAGGAGAAAATACCGCATCAG |
|  | rec-hphrev | ACTTTATGCTTCCGGCTCGTATGTT |
|  | p-5’ | ATCTACTGACACAGCCAAATTACCG |
|  | p-3’ | GAATGAACAACGGTGGATAAATCTT |
| **Primers used in the identification of deletion mutant of genes *sapl*** | | |
| *sapl1* | A-p1 | CGAACAGTTATTTTCTGCATCATCA |
|  | A-p2 | TTCCTCACCGCCTCTTATGCT |
| *sapl2* | B-p1 | TTTTTCTCTTGATCATCATCTTCTC |
|  | B-p2 | CCGTCCCAAATTAGCTCAGTT |
| *sapl3* | C-p1 | TTTTTCTCTTGATCATCATCTTCTC |
|  |  |  |
|  | C-p2 | CCGTCCCAAATTAGCTCAGTT |
| *sapl4* | D-p1 | TTCTTTCTGAATCCTTGTCCTCTCT |
|  | D-p2 | TGTGGCGAAAACGGATAGGT |
| *hph* | hph-p1 | ATGAAAAAGCCTGAACTCACC |
|  | hph-p2 | CTATTCCTTTGCCCTCGGAC |

**Supplementary Table S2.** Primary extracellular protein with ammonium sulfate as source

| Gene | # PSMs | MW [kDa] | calc. pI |
| --- | --- | --- | --- |
| GME2276_g | 282 | 42.3 | 4.48 |
| GME2936_g | 269 | 99.2 | 4.77 |
| GME2698_g | 152 | 23.3 | 4.25 |
| GME2461_g | 133 | 39.9 | 4.73 |
| GME4480_g | 104 | 38.6 | 4.87 |
| GME1365_g | 103 | 32.8 | 4.65 |
| GME3216_g | 99 | 63.7 | 4.82 |
| GME2992_g | 69 | 57.5 | 4.48 |
| GME4_g | 92 | 93.4 | 6.70 |
| GME1246_g | 74 | 58.4 | 4.70 |
| GME2540_g | 61 | 91.8 | 5.40 |
| GME587_g | 53 | 49.5 | 4.69 |
| GME4064_g | 57 | 43.3 | 4.91 |
| GME2583_g | 46 | 69.3 | 5.11 |
| GME2647_g | 52 | 44.8 | 5.10 |
| GME2632_g | 47 | 24.1 | 6.74 |
| GME3481_g | 38 | 43.7 | 4.54 |
| GME161_g | 39 | 49.6 | 4.69 |
| GME2999_g | 37 | 43.3 | 4.44 |
| GME3460_g | 35 | 55.8 | 4.65 |
| GME1686_g | 35 | 58.9 | 4.68 |
| GME3358_g | 26 | 54.5 | 4.91 |
| GME2207_g | 29 | 43.9 | 4.69 |
| GME4116_g | 19 | 94.2 | 5.63 |

**Supplementary Table S3.** Primary extracellular protein with yeast extract as source

| Gene | # PSMs | MW [kDa] | calc. pI |
| --- | --- | --- | --- |
| GME2276_g | 359 | 42.3 | 4.48 |
| GME2936_g | 438 | 99.2 | 4.77 |
| GME2992_g | 204 | 57.5 | 4.48 |
| GME161_g | 197 | 49.6 | 4.69 |
| GME3216_g | 150 | 63.7 | 4.82 |
| GME2698_g | 116 | 23.3 | 4.25 |
| GME3356_g | 126 | 33.0 | 6.52 |
| GME2999_g | 105 | 43.3 | 4.44 |
| GME1246_g | 73 | 58.4 | 4.70 |
| GME3481_g | 63 | 43.7 | 4.54 |
| GME3460_g | 60 | 55.8 | 4.65 |
| GME4064_g | 61 | 43.3 | 4.91 |
| GME4_g | 59 | 93.4 | 6.70 |
| GME2540_g | 72 | 91.8 | 5.40 |
| GME587_g | 59 | 49.5 | 4.69 |
| GME329_g | 52 | 45.5 | 4.78 |
| GME2583_g | 45 | 69.3 | 5.11 |
| GME2647_g | 43 | 44.8 | 5.10 |
| GME823_g | 32 | 34.5 | 4.54 |
| GME1317_g | 31 | 25.9 | 5.19 |
| GME3358_g | 17 | 54.5 | 4.91 |
| GME317_g | 18 | 74.7 | 4.34 |
| GME3803_g | 22 | 42.7 | 4.50 |
| GME1686_g | 17 | 58.9 | 4.68 |
